# Discrimination of Milks with a Multisensor System Based on Layer-by-Layer Films

**DOI:** 10.3390/s18082716

**Published:** 2018-08-18

**Authors:** Coral Salvo-Comino, Celia García-Hernández, Cristina García-Cabezón, Maria Luz Rodríguez-Méndez

**Affiliations:** 1 Group UVaSens, Engineers School, Universidad de Valladolid, 47011 Valladolid, Spain; coraldeugena@hotmail.com (C.S.-C.); celiagarciahernandez@gmail.com (C.G.-H.); anacrigar@gmail.com (C.G.-C); 2 BioecoUVA Institute, Engineers School, Universidad de Valladolid, 47011 Valladolid, Spain

**Keywords:** electronic tongue, milk, galactose, phthalocyanine, chitosan, ionic liquid, LbL sensor

## Abstract

A nanostructured electrochemical bi-sensor system for the analysis of milks has been developed using the layer-by-layer technique. The non-enzymatic sensor [CHI+IL/CuPc^S^]_2_, is a layered material containing a negative film of the anionic sulfonated copper phthalocyanine (CuPc^S^) acting as electrocatalytic material, and a cationic layer containing a mixture of an ionic liquid (IL) (1-butyl-3-methylimidazolium tetrafluoroborate) that enhances the conductivity, and chitosan (CHI), that facilitates the enzyme immobilization. The biosensor ([CHI+IL/CuPc^S^]_2_-GAO) results from the immobilization of galactose oxidase on the top of the LbL layers. FTIR, UV–vis, and AFM have confirmed the proposed structure and cyclic voltammetry has demonstrated the amplification caused by the combination of materials in the film. Sensors have been combined to form an electronic tongue for milk analysis. Principal component analysis has revealed the ability of the sensor system to discriminate between milk samples with different lactose content. Using a PLS-1 calibration models, correlations have been found between the voltammetric signals and chemical parameters measured by classical methods. PLS-1 models provide excellent correlations with lactose content. Additional information about other components, such as fats, proteins, and acidity, can also be obtained. The method developed is simple, and the short response time permits its use in assaying milk samples online.

## 1. Introduction

The dairy industry is under constant renovation, to adapt itself to new technologies and consumer demands, as well as to the strict legislation of the sector. Due to these challenges, the industry needs to develop new improved technologies for the quality control. Lactose intolerance or obesity might cause consumers to turn to lactose-free milk and low fat milk. When milk is received from the farm, it is pasteurized and standardized to the desired milk fat percentage (skim, 1%, 2%, or 3.5%). Lactase enzyme is then added to remove the lactose. Lactase hydrolyzes the disaccharide lactose into constituent galactose and glucose monomers that can be absorbed across the intestinal mucosa. Lactose, glucose, and galactose contents in dairy products are determined by conventional techniques, such as HPLC-MS, GC-MS, colorimetry, potentiometry, gravimetry, and polarimetry, among others [1,2]. However, these methods require long times and large investments, and cannot be used in field analysis. This situation has stimulated the interest in developing electrochemical sensor-based methodologies for milk analysis, and many examples can be found in the literature [3,4,5,6,7]. Electrochemical biosensors are particularly interesting, because they show high selectivity and can play a fundamental role in improving the quality control of milk [8,9].

During the last years, a variety of nanostructured electrodes have been developed. The layer-by-layer (LbL) method is one of the most suitable methods to obtain nanostructured sensors and biosensors, owing to its efficient control on the architecture and its inherent simplicity and low cost [10]. Besides, a large choice of sensing materials can be deposited using the LbL technique. Moreover, hybrid structures can be developed, combining different materials in the layered structure that can improve the final sensing properties. Finally, it is an advantageous method to immobilize enzymes in a biomimetic environment [11,12,13,14,15].

Phthalocyanines have been widely used as sensing materials in electrochemical sensors due to their chemical reactivity, their excellent electrocatalytic properties, as well as the capability to form LbL nanostructured films [11,16]. The LbL technique can be used to combine the water soluble anionic metal tetrasulfonated phthalocyanines (MPc^S^) with other cationic materials, to obtain hybrid layers with improved catalytic properties [17]. One of the drawbacks of phthalocyanines is their low conductivity. It can be expected that a hybrid material formed by a combination of a phthalocyanine with a highly conductive material can improve the sensitivity of the sensors. Ionic liquids (IL) are excellent candidates to do this task, due to their high ionic conductivity. A large choice of ILs are available with different solubilities and ionic charges [18]. Finally, it has been demonstrated that chitosan (CHI), a cationic polyaminosaccharide, acts to increase the active surface. Taking into account these premises, it can be expected that an LbL film combining CHI, IL, and CuPc^S^ can show an increased sensitivity towards a variety of compounds [19].

The enzyme galactose oxidase (GAO) is an oxidoreductase enzyme of interest in the analysis of dairy products, because it can be used to detect galactose in lactose-free dairy products. GAO is able to oxidize D-galactose, giving the corresponding aldehyde (D-galactohexodialdose) accompanied by the reduction of O_2_ to H_2_O_2_. The activity of GAO can be improved by using an appropriate matrix and a suitable electron mediator [20,21,22]. Phthalocyanines have been demonstrated to be efficient electron mediators, and CHI can be an ideal support for enzyme immobilization through the amino groups. For these reasons, it can be foreseen that the performance of a GAO biosensor can be improved using a matrix where both materials interact with the enzyme.

Electronic tongues (e-tongues) consist of an array of low-selective sensors combined with advanced mathematical procedures for signal processing based on pattern recognition and/or multivariate data analysis. The sensing elements have partial specificity, and they respond to a range of compounds present in the sample, rather than to a specific chemical species. The ensemble of signals provided by the whole array provides a fingerprint of each sample, and can be used to discriminate samples using PCA [23,24,25].

E-tongues based on different types of sensors have been used to analyze the quality of milks [26,27] to monitor changes in quality during storage [28,29], to classify fermented milks [30], and to detect adulterations [31] or off-flavors in the raw milk [32]. Usually e-tongue systems provide global information about the samples and are used for classification purposes using principal component analysis (PCA). However, using the adequate mathematical models, it is possible to establish correlations between the signals produced by the multisensor system and data obtained with classical analysis. In these cases, after the appropriate training, e-tongues can be used to obtain information about specific components [33,34,35].

Sensors are at the heart of electronic tongues, and much research is being carried out to improve the performance of these sensors. Nanostructured films can be an alternative because the LbL technique can be used to prepare composite materials with improved properties. They can be simultaneously excellent substrates for enzyme immobilization. Some examples of e-tongues, using Langmuir–Blodgett or layer-by-layer films, have been published [36,37], but much work needs to be carried out to develop new sensors with better operation characteristics.

In this work, LbL films will be developed, in order to obtain a two-sensor system able to classify milk samples according to their lactose content. The first sensor will be a general purpose sensor formed by LbL layers alternating an electrocatalytic material (the anionic copper sulfonate phthalocyanine, CuPc^S^), and a cationic layer combining a biomaterial able to increase the active surface (chitosan, CHI) with an ionic liquid (IL) that would to increase the conductivity and the efficiency of the electron transfer. The second sensing unit will be a biosensor specific for the detection of galactose, where the enzyme GAO will be deposited on the LbL film that this time will work as the electron mediator, and will take advantage of the capability of CHI to link enzymes.

## 2. Materials and Methods

Chitosan (CHI), 1-butyl-3-methyliimidazolium tetrafluoroborate (IL), copper phthalocyanine-3,4′,4″,4′′′-tetrasulfonic acid tetrasodium salt (CuPc^S^), and all other reagents were purchased from Sigma-Aldrich. Galactose oxidase, from *Aspergillus niger* (activity of 168,400 U·mg^−1^) was also purchased from Sigma-Aldrich (Darmstad, Germany) Deionized water with resistivity 18.2 MΩ·cm, was obtained from a Milli-Q (Merck-Millipore, Darmstad, Germany) system.

Layer-by-layer films were deposited using a ND-R Rotary Coater device. They were obtained by depositing bilayers of a mixture of CHI and IL (cationic species) and CuPc^S^ (anionic species) to obtain [CHI+IL/CuPc^S^]_n_. For this purpose, ITO glass was incubated in a 1:1 mixture of a 2 mmol·L^−1^ solution of IL and 2 mg·mL^−1^ of CHI prepared in acetic acid 0.3% (*v*/*v*) for 5 min. The film was then washed in distilled water (5 s), and immersed for 5 min in a 0.05 mg·mL^−1^ solution of CuPc^S^. The cycle was repeated twice to obtain a bilayer.

**Figure 1:**
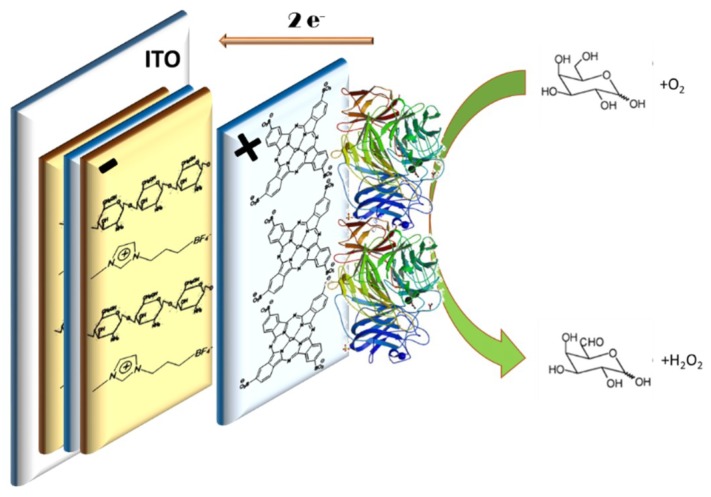
Structure of the [CHI+IL/CuPc^S^]_2_-GAO biosensor. Scheme of the reaction.

The biosensor was prepared by depositing a layer of GAO on the top of the LbL film to obtain [CHI+IL/CuPc^S^]_n_-GAO, using 50 µL of a GAO solution (5 mg·mL^−1^) (in 0.01 mol·L^−1^ phosphate buffer, pH 7). After drying, GAO was crosslinked with vapors of a glutaraldehyde solution 2.5% (*v*/*v*) in 0.01 mol·L^−1^ phosphate buffer, pH 7. Finally, films were washed with phosphate buffer to remove the unbound enzyme. Figure 1 shows the structure of the prepared films.

Films deposited on quartz were characterized by UV–vis using a Shimadzu UV-2600 spectrophotometer. FTIR characterization was carried out in films deposited on ZnS using a FTIR 6600 Jasco spectrophotometer. Spectra were recorded from 400 to 4000 cm^−1^, at a resolution of 4 cm^−1^ and 1000 scans. The topography of LbL films deposited on mica, was analyzed by atomic force microscopy (AFM) in a NanoScope IIIa operated in tapping mode. Voltammograms were registered in a potentiostat/galvanostat PGSTAT128 (Autolab Metrohm, Utrecht, The Netherlands) in a three-electrode electrochemical cell (50 mL) using the LbL films deposited on ITO glass as the working electrode. Ag|AgCl|KCl was used as the reference electrode, and a platinum sheet (2 cm^2^) as the counter electrode.

Four different milk samples with different fat and lactose contents were obtained from a local supplier. Two samples were whole milk (with 3.5% fat) with lactose (samples Wlac_1_ and Wlac_2_), and two samples were semi-skimmed (with 1.8% fat) and lactose-free (samples SSnolac_1_ and SSnolac_2_). Samples were analyzed without any prior pretreatment, the same day that the Tetra Brik pack was opened. The spoilage of milks was evaluated by analyzing the samples four days after the pack was opened (samples kept at room temperature). 

All the samples were measured five times with each sensor. The multivariate data analysis was performed by using MATLAB R2014b (The Mathworks Inc., Natick, MA, USA). The principal component analysis (PCA) was carried out using, as input data source, pre-processed voltammograms obtained by the adaptation of a data reduction technique based on predefined response “bell-shaped-windowing” curves called “kernels” [38,39]. Using this method, voltammograms were multiplied by 10 smooth, bell-shaped windowing functions, defined as

(1) $$K_{i}\left(V_{j}\right)=\frac{1}{1+{\left(\frac{V_{j}-c_{i}}{a_{i}}\right)}^{2b_{i}}},$$

where *a_i_*, *b_i_*, and *c_i_* define the width, shape, and center of the different windowing functions, *K_i_*. The obtained data were integrated with respect to voltage. All the classification models were subjected to full cross-validation by means of the “leave-one-out” method. Mathematical correlations between the signals obtained using the sensors and data obtained by classical chemical analysis were established using partial least squares (PLS-1) models.

## 3. Results and Discussion

### 3.1. Structural Characterization of the Sensors

**Figure 2:**
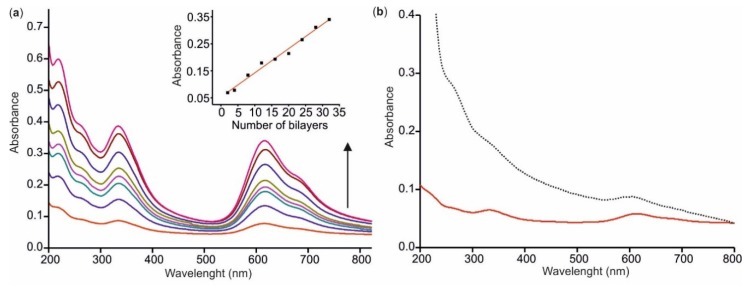
(**a**) UV–vis spectra of [CHI+IL/CuPc^S^]_n_ films with increasing number of layers (*n* = 2, 4, 8, 12, 16, 20, 24, 28, 32). The inset shows the correlation between absorbance at 617 nm and the number of layers; (**b**) comparison of the spectra of [CHI+IL/CuPc^S^]_2_ (solid red line) and of [CHI+IL/CuPc^S^]_2_-GAO (dotted black line).

The LbL nanostructured films were characterized by UV–vis spectroscopy (Figure 2). As expected, spectra showed a Soret band at 335 nm corresponding to the (HOMO)_a2u_-(LUMO)_eg_ transition. Two intense Q bands appeared at 680 nm and 617 nm. The band at 680 nm was associated to the presence of CuPc^S^ monomers, and the band at 617 nm was due to the presence of dimers formed by the π–π* stacking of the phthalocyanine macrocycles [40]. Figure 2a also shows the UV–vis spectra obtained when increasing the number of layers (from 2 to 32 bilayers). The inset established that the absorbance measured at 617 nm showed a linear relationship with the number of layers, with a correlation coefficient of 0.985, confirming the good quality of the films. Figure 2b compares the electronic absorption spectra of the [CHI+IL/CuPc^S^]_2_ sensor and the [CHI+IL/CuPc^S^]_2_-GAO biosensor. Besides the peaks already described, the spectrum of the biosensor showed the typical band at 280 nm produced by the presence of the enzyme.

**Figure 3:**
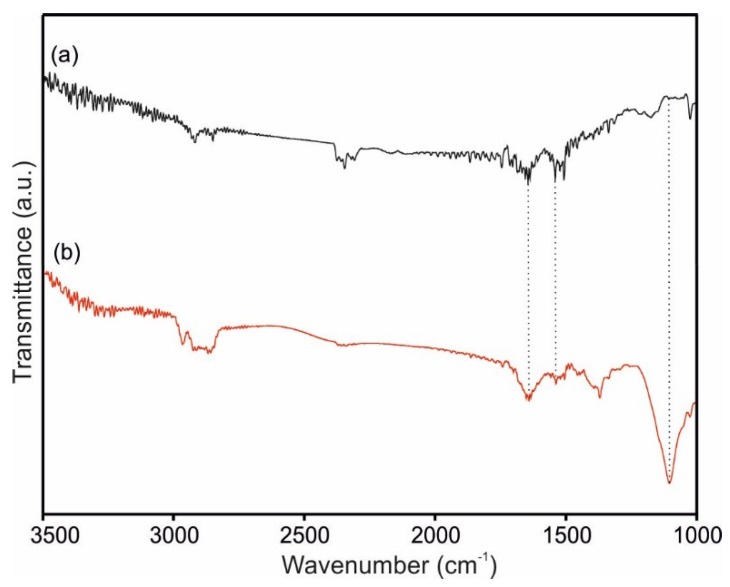
FTIR spectra of LbL for (**a**) [CHI+IL/CuPc^S^]_8_ films and (**b**) [CHI+IL/CuPc^S^]_8_-GAO films.

FTIR spectra were registered to confirm the efficient transfer of the LbL layers. In Figure 3, the spectra of an 8 bilayer LbL film of [CHI+IL/CuPc^S^]_8_, and of a [CHI+IL/CuPc^S^]_8_-GAO biosensor, are compared. The assignments for the main peaks are given in Table 1. The spectrum of the [CHI+IL/CuPc^S^]_8_ showed the characteristic peaks of all three components. For instance, the band at 1027 cm^−1^ was related to the isoindole deformation and aza stretching of CuPc^S^, and the band at 1174 cm^−1^ was related to S=O symmetric stretching vibrations in sulfonic groups [41]. Characteristic bands of CHI appeared at 3400 cm^−1^, corresponding to the OH stretching vibrations, and 1367 cm^−1^, due to the C–H–OH bending and CH_2_OH stretching, respectively [42]. The main bands produced by the IL appeared at 1647 cm^−1^, corresponding to the C=C and C=N bonds (C–N stretching), and at 1078 cm^−1^, related to C–H in-plane deformation [43].

The [CHI+IL/CuPc^S^]_8_-GAO film showed not only the peaks already mentioned, but it also showed the typical amide I and amide II bands of the enzyme, that appeared at 1650 cm^−1^ and 1543 cm^−1^, respectively. An intense band appeared at 1107 cm^−1^, due to the hydroxyl groups of the enzyme [44,45]. These features confirmed that GAO was immobilized onto the surface of the LbL matrix. 

The band assignment confirmed the correct adsorption of the bilayers onto the electrode surface.

**Figure 4:**
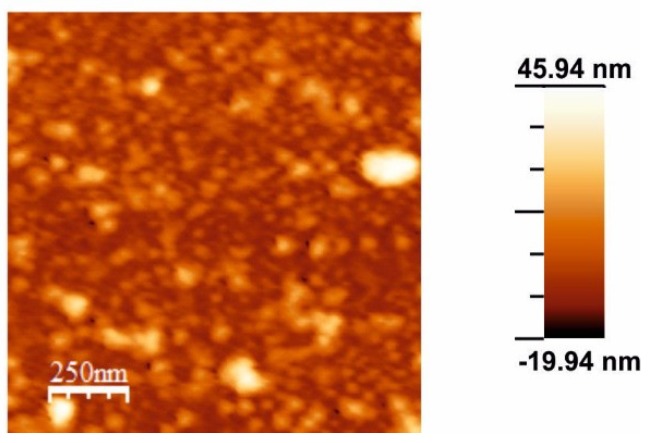
AFM topographic 2D images of a LbL film formed by [CHI-IL/CuPc^S^]_12_ film.

AFM images of the surface of the LbL [CHI+IL/CuPc^S^]_12_ (Figure 4) showed smooth surfaces with RMS of 7.7 nm. AFM images of the [CHI+IL/CuPc^S^]_12_-GAO film showed a very rough globular surface that was a reflection of the presence of the enzyme adsorbed on the surface.

### 3.2. Electrochemical Characterization

The sensing properties of [CHI+IL/CuPc^S^]_2_ and [CHI+IL/CuPc^S^]_2_-GAO deposited on ITO glass were evaluated by cyclic voltammetry. Cycles were carried out from −800 to +1200 mV at scan rate of 100 mV·s^−1^ in a solution of galactose, 10^−4^ mol·L^−1^ in 0.01 mol·L^−1^ phosphate buffer, pH 7.

**Figure 5:**
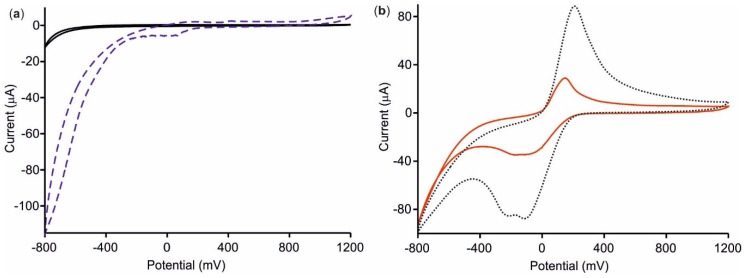
Cyclic voltammograms of (**a**) bare ITO (solid black line) and [CHI+IL/CuPc^S^]_2_ film (dashed blue line), and (**b**) ITO-GAO (solid red line) and [CHI+IL/CuPc^S^]_2_-GAO (dotted black line), immersed in galactose 10^−4^ mol·L^−1^ in 0.01 mol·L^−1^ phosphate buffer, pH 7. Scan rate 100 mV·s^−1^.

To study the co-amplification effect of the CHI, IL, and CuPc^S^ on the sensing properties of [CHI+IL/CuPc^S^]_2_, the response of the film towards galactose was compared with that obtained using a bare ITO electrode. As observed in Figure 5a, the bare ITO glass immersed in galactose did not show redox processes. ITO, modified with the [CHI+IL/CuPc^S^]_2_ LbL films, showed a redox pair with an E_1/2_ of ca. 0.1 V, produced by the oxidation/reduction of galactose. An intense cathodic peak at −0.8 V, corresponding to the decomposition of water, was also observed. Voltammograms were reproducible, and the variation coefficient, measured as variation of the peak intensity in 5 consecutive measurements, was 3.5%. The increase in the intensity of the signals with respect to bare ITO was probably due to the electrocatalytic effect of the CuPc^S^ and the enhanced electrical conductivity of the IL. In fact, this amplification was not observed when individual components were tested, indicating that magnification was due to the simultaneous effect of both materials.

The electrochemical responses of the biosensor [CHI+IL/CuPc^S^]_2_-GAO and the electron mediator effect of the LbL layer were also tested in galactose. Results were compared with those obtained using GAO deposited directly on the ITO glass (Figure 5b). When the enzyme was immobilized directly on the ITO surface, the response towards galactose showed a quasi-reversible redox pair formed by a cathodic wave at −125 mV due to the reduction of the Cu present in the coenzyme, from Cu(II) to Cu(I), that accompanied the oxidation of galactose. The anodic wave at 150 mV was also observed due to the oxidation from Cu(I) to Cu(II) [22].

The response of [CHI+IL/CuPc^S^]_2_-GAO, demonstrated that the presence of the LbL layers increased, drastically, the intensity of the redox peaks from 30 µA to 90 µA. It was inferred that the LbL film could effectively improve the performance of the biosensor through the good immobilization promoted by CHI, and the excellent electron-mediator properties of CuPc^S^ and IL, resulting in the amplification of the current response. Voltammograms were also reproducible, and the variation coefficient was 6%.

Detection limits were calculated by chronoamperometry in galactose solutions from 10^−4^ mol·L^−1^ to 10^−9^ mol·L^−1^ (in 0.01 mol·L^−1^ phosphate buffer, pH 7). From the calibration curves registered in the reduction peaks, a LOD of 3.6 × 10^−9^ mol·L^−1^ for the [CHI+IL/CuPc^S^]_2_ sensor and 9.4 × 10^−11^ mol·L^−1^ for the [CHI+IL/CuPc^S^]_2_-GAO biosensor were determined. Experimental results show that double amplification, which is based on the catalysis of the LbL films and the enzymatic effect of galactose oxidase, can remarkably increase the assay sensitivity.

### 3.3. Response of the Multisensor System to Milk Samples. Discrimination and Correlations with Chemical Parameters

**Figure 6:**
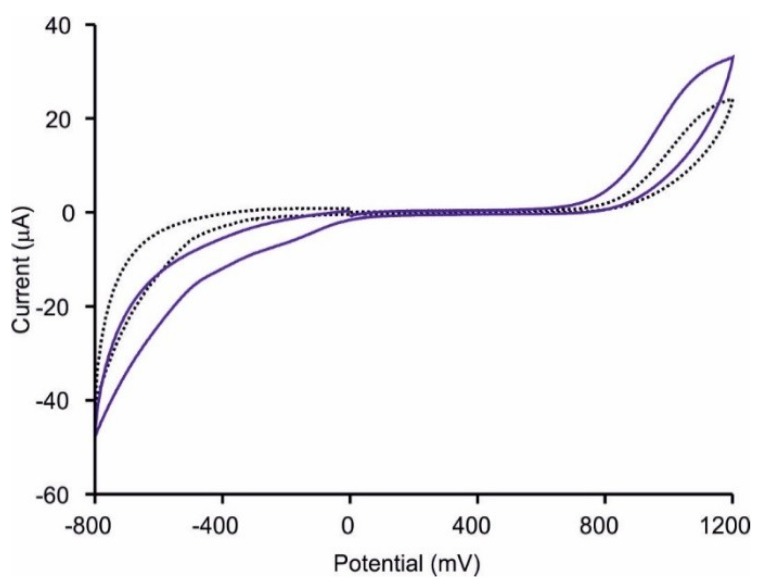
Cyclic voltammograms of [CHI-IL/CuPc^S^]_2_ (dotted black line), and for [CHI-IL/CuPc^S^]_2_-GAO (solid blue line) sensor immersed in sample SSnolac_2_. Scan rate 100 mV·s^−1^.

A multisensor system was formed by combining [CHI+IL/CuPc^S^]_2_ and [CHI+IL/CuPc^S^]_2_-GAO sensors with pattern recognition software. Cyclic voltammetry was carried out from −800 to +1200 mV at a scan rate of 100 mV·s^−1^ in milk samples without any pretreatment. Each sensor showed distinct responses when immersed in different types of milks. For instance, as observed in Figure 6, when [CHI+IL/CuPc^S^]_2_-GAO was immersed in milk with and without lactose, voltammograms were different. In both cases, a broad anodic peak at ca. +900 mV was observed. The cathodic wave showed an intense peak at −900, and in the case of the sample with lactose, a shoulder at −300 mV was also observed. Due to the complexity of milk, it is difficult to assign the origin of the peaks, except for the peak at −300 mV that can be attributed to the presence of galactose detected by the GAO, corroborating the role of the enzyme.

**Figure 7:**
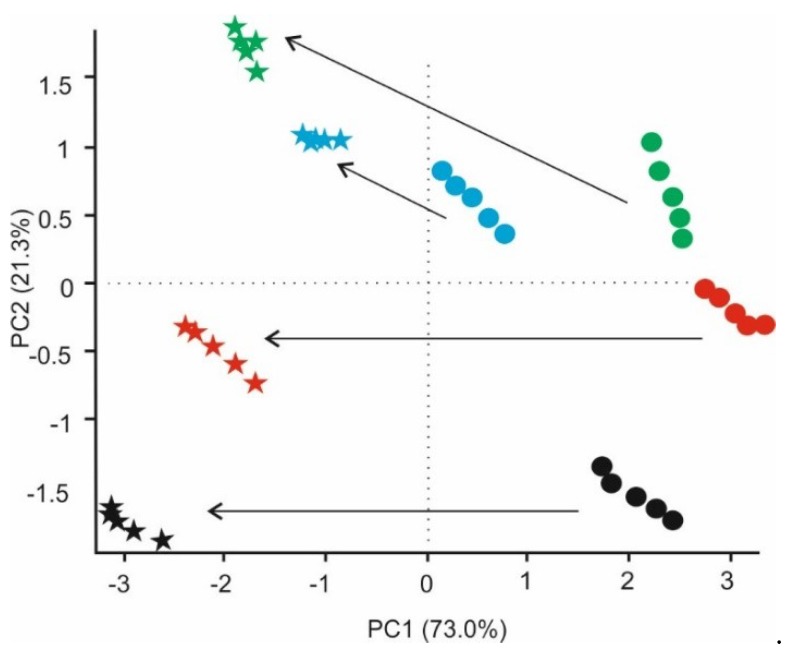
PCA plots for milk samples obtained from voltammetric responses in milks of sample Wlac_1_: first day (●), fourth day (★); sample Wlac_2_: first day (●), fourth day (★); sample SSnolac_1_: first day (●), fourth day (★); and sample SSnolac_2_: first day (●), fourth day (★).

Once all milk samples were measured in quintuplicate, voltammograms were pretreated using the kernel method, and 10 variables per senor were obtained. These data were used as the input variable in PCA analysis. The PCA scores plot is presented in Figure 7. The two first components captured 94.3% of the information (PC1 73%, PC2 21.3%). The e-tongue showed well-defined and separated clusters for each type of milk: whole milks with lactose appear in the lower part of the graph at negative values of PC2 axis, whereas, semi-skimmed milk without lactose appeared in upper part of the diagram in the positive PC2 region. Hence, PC2 is the component that contains the information related to the presence of lactose. When measures were repeated four days after opening the Tetra Brik package, clusters shifted to the left side of the diagram, confirming the capability of the system to detect spoilage.

**Figure 8:**
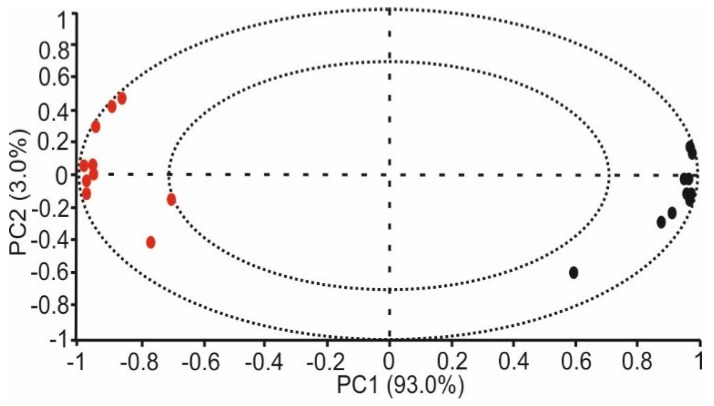
Loading plot of PCA performed from milk samples using the sensor [CHI+IL/CuPc^S^]_2_ and the biosensor [CHI+IL/CuPc^S^]_2_-GAO.

Figure 8 showed the contribution of each variable of the bi-sensor system in a loadings plot. The graph was built with ten variables per sensor (represented in black circles) that were originated by sensor [CHI+IL/CuPc^S^]_2_, and variables represented in red circles were obtained from the voltammetric responses of the biosensor [CHI+IL/CuPc^S^]_2_-GAO. As observed in the loading plot, all the variables showed correlation loadings higher than 0.5. The important cross-selectivity achieved by the sensors was corroborated by that fact that the information provided by both sensors appeared in opposite regions of the plot. In fact, the sensor [CHI+IL/CuPc^S^]_2_ had an important influence in the positive region of the PC1, whereas the information provided by the [CHI+IL/CuPc^S^]_2_-GAO biosensor was located in the negative region of PC1.

In the last part of the work, a PLS-1 multiparametric model was established to find correlations between the results obtained with the sensor system, with data obtained from chemical analysis (Table 2). The most common parameters usually measured in quality control were included in the study: fat content (Fat), protein content, lactose, FPD (freezing point depression), urea, acidity, ESM (fat free dry matter), somatic cells, and UV–vis 1:100. These parameters were measured following standard methods [46].

Table 3 shows the statistical parameters for the PLS-1 regression for calibration and validation (cross-validation). The root mean square errors and the correlation coefficients at calibration (RMSEC) and validation (RMSEV) are shown in Table 3. Rc (and R_V_) are correlation coefficients of calibration (and validation). LV indicates the number of latent variables used in the calculations.

**Figure 9:**
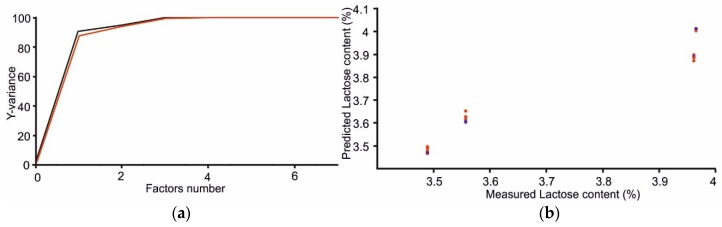
(**a**) Explained variance of lactose content in function of the number of latent variables; (**b**) linear correlation between lactose measured and lactose predicted.

Due to the presence of galactose oxidase, excellent linear relationships were found between the responses provided by the multisensor system and the lactose content (Figure 9 and Table 3). 

It is important to bear in mind that other components present in milk can affect the electrochemical response. For instance, acidity can modify the response of the enzyme in the [CHI+IL/CuPc^S^]_2_-GAO sensor, or can alter the response of the [CHI+IL/CuPc^S^]_2_ device at negative voltages_._ The presence of fats and proteins can modify the electrical conductivity. Thus, the array can be used to assess not only the lactose content, but it can also be used to simultaneously determine other components. 

Good relationships were found between the responses provided by the sensors and parameters related to the protein content (protein, urea, cells), acidity, fat content, and with the color (UV–vis 1:100), with *R*^2^ close to 1 (both in calibration and validation), low residual errors, and also a low number of latent variables.

## 4. Conclusions

A multisensor system, formed by LbL sensors, has been successfully developed and used to analyze milks. It has been demonstrated that the combination in a LbL film of CuPc^S^ (acting as electrocatalytic material), IL (that enhances the conductivity), and CHI (that increases the active surface), show a synergistic effect that improves the response of the [CHI+IL/CuPc^S^]_2_ sensor. In turn, the same combination of materials is an efficient electron mediator in the [CHI+IL/CuPc^S^]_2_-GAO sensor.

A multisensory system formed by the combination of the two nano-layered sensors has been successfully used to discriminate milk samples with different lactose content, and to detect the spoilage. Using PLS-1, it is possible to assess lactose, fat, protein content, and acidity. The method is simple and fast, and could be further expanded to detect other milk components.

## Figures and Tables

**Table 1:** Assignments for the FTIR spectra of LbL films in Figure 3.

Film Material	Peak Position (cm^−1^)	Assignment
CuPc^S^	1027	υ(C–N) stretching in pyrrole vibration
	1174	S=O symmetric stretching vibration
CHI	1367	CH_2_OH stretching
	3400	OH stretching vibration
IL	1078	C–H deformation in plane
	1647	C=C and C=N stretching
GAO	1107	Hydroxyl group
	1540	Amide II, NH bending, and CN stretching
	1650	Amide I, C=O stretching, and CN stretching

**Table 2:** Chemical parameters measured in milks.

ID	Lactose	Fat	Proteins	FPD	Urea	Acidity	ESM	Cells	UV–vis
**Wlac1**	4.87	3.52	3.22	8.842	242	13.8	8.842	17	3.459
**WWlac2**	4.86	3.53	3.26	9.009	262	14.4	9.009	32	3.493
**WSSnolac1**	3.32	1.80	3.40	7.325	619	11.4	7.325	25	3.108
**WSSnolac2**	3.14	1.84	3.30	7.195	594	11.6	7.195	15	3.065

**Table 3:** Results of the PLS-1 analysis.

Parameter	R^2^c ^(a)^	RMSE_C_^(b)^	R^2^_V_^(c)^	RMSE_V_^(d)^	LV ^(e)^
**WFat**	0.9243	0.2345	0.9050	0.2770	2
**WProteins**	0.9494	0.0151	0.9280	0.0190	1
**WLactose**	0.9439	0.1943	0.9338	0.2220	2
**WFDP**	0.9467	0.0820	0.9270	0.0101	1
**WUrea**	0.9206	50.0362	0.9031	58.1922	2
**WAcidity**	0.9232	0.3655	0.8939	0.4523	2
**WESM**	0.9416	0.2020	0.9230	0.2443	2
**WCells**	0.9976	0.3295	0.9867	0.8603	3
**WUV–vis**	0.9038	0.0607	0.8751	0.0728	1

^(a)^ Squared correlation coefficient in calibration; ^(b)^ root mean square error of calibration; ^(c)^ squared correlation coefficient in validation; ^(d)^ root mean square error of validation; ^(e)^ latent variables.
